# Understanding multivariate brain activity: Evaluating the effect of voxelwise noise correlations on population codes in functional magnetic resonance imaging

**DOI:** 10.1371/journal.pcbi.1008153

**Published:** 2020-08-18

**Authors:** Ru-Yuan Zhang, Xue-Xin Wei, Kendrick Kay

**Affiliations:** 1 Shanghai Key Laboratory of Psychotic Disorders, Shanghai Mental Health Center, School of Medicine, Shanghai Jiao Tong University, Shanghai, China; 2 Institute of Psychology and Behavioral Science, Shanghai Jiao Tong University, Shanghai, China; 3 Center for Magnetic Resonance Research, Department of Radiology, University of Minnesota, Minneapolis, Minnesota, United States of America; 4 Department of Neuroscience, Center for Perceptual Systems, Institute for Neuroscience, University of Texas at Austin, Texas, United States of America; Oxford University, UNITED KINGDOM

## Abstract

Previous studies in neurophysiology have shown that neurons exhibit trial-by-trial correlated activity and that such noise correlations (NCs) greatly impact the accuracy of population codes. Meanwhile, multivariate pattern analysis (MVPA) has become a mainstream approach in functional magnetic resonance imaging (fMRI), but it remains unclear how NCs between voxels influence MVPA performance. Here, we tackle this issue by combining voxel-encoding modeling and MVPA. We focus on a well-established form of NC, tuning-compatible noise correlation (TCNC), whose sign and magnitude are systematically related to the tuning similarity between two units. We show that this form of voxelwise NCs can improve MVPA performance if NCs are sufficiently strong. We also confirm these results using standard information-theoretic analyses in computational neuroscience. In the same theoretical framework, we further demonstrate that the effects of noise correlations at both the neuronal level and the voxel level may manifest differently in typical fMRI data, and their effects are modulated by tuning heterogeneity. Our results provide a theoretical foundation to understand the effect of correlated activity on population codes in macroscopic fMRI data. Our results also suggest that future fMRI research could benefit from a closer examination of the correlational structure of multivariate responses, which is not directly revealed by conventional MVPA approaches.

## Author summary

Noise correlation (NC) is the key component of multivariate response distributions and thus characterizing its effects on population codes is the cornerstone for understanding probabilistic computation in the brain. Despite extensive studies of NCs in neurophysiology, little is known with respect to their role in functional magnetic resonance imaging (fMRI). We characterize the effect of voxelwise NC by building voxel-encoding models and directly quantifying the amount of information in simulated multivariate fMRI data. In contrast to the detrimental effects of NC implied in neurophysiological studies, we find that voxelwise NCs can enhance information codes if NC is sufficiently strong. Our work highlights the important role of noise correlations in decipher population codes using fMRI.

## Introduction

Understanding how neural populations encode information and guide behavior is a central question in modern neuroscience. In a neuronal population, many units exhibit correlated activity, and this likely reflects an important feature of information coding in the brain. In computational neuroscience, researchers have investigated the relationship between *signal correlation* (SC), referring to the similarity between the tuning functions of two neurons, and *noise correlation* (NC), referring to the correlation between two neurons’ trial-by-trial responses evoked by repetitive presentations of the same stimulus [1–3].

Previous studies in neurophysiology have discovered that neurons that share similar tuning functions (i.e., a positive SC) also tend to have a weak positive NC, a pervasive phenomenon across several brain regions [4–11]. In this paper, we denote this type of NC as *tuning-compatible noise correlation* (TCNC) because the sign and the magnitude of the NC are systematically related to the SC between a pair of neurons. A bulk of theoretical and empirical work has shown that NCs have a substantial impact on population codes. For example, the seminal study by Zohary, Shadlen [12] demonstrated that TCNCs limit the amount of information in a neural population as the noise is shared by neurons and cannot be simply averaged out. Later on, researchers realized that this detrimental effect of TCNC is mediated by other factors, such as the form of NC, heterogeneity of tuning functions, and its relevance to behavior [2, 13–16].

**Fig 1:**
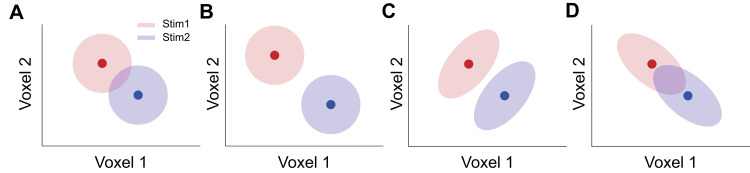
A two-voxel scenario in MVPA. The pool consists of two responsive voxels and the two color disks represent the trial-by-trial response distributions evoked by two different stimuli. Panel A illustrates the original state of the population responses. Decoding performance can be improved via either a bigger separation of the mean population response (panel B) or changes in the covariance structure (panel C). Representational structures in panels B and C indicate improved population codes but have distinct underlying mechanisms. Panel D illustrates that certain covariance changes can worsen decoding.

The study of NCs in the brain has been historically impeded by technical barriers to measuring simultaneously the activity of many neurons in neurophysiological experiments. In contrast, functional magnetic resonance imaging (fMRI) naturally measures the activity of many neural populations throughout the entire brain. Imaging scientists often use multivariate pattern analysis (MVPA) to assess the accuracy of population codes [17, 18]. However, above-chance decoding performance in MVPA does not specify the detailed representational structure underlying multivariate voxel responses. For example, Fig 1 illustrates a simple two-voxel scenario in multivariate decoding. The decoding accuracy in the original state (Fig 1A) can be improved (e.g., by attention, learning) via either the further separation of mean responses (Fig 1B) or the changes to the covariance geometry (Fig 1C). This example highlights the impact of the shape of the response distribution on population codes and these effects cannot be easily disentangled by the conventional MVPA approach [19].

The magnitude and the structure of NCs in fMRI data still remain largely unknown. It has been shown that NCs influence MVPA accuracy and that certain types of classifiers can compensate for NCs [20]. But the precise nature of NCs has not yet been thoroughly characterized. There have been a few recent investigations of NCs. A study by Ryu and Lee [21] evaluated the impact of three factors—retinotopic distance, cortical distance, and tuning similarity—on voxelwise NCs in early visual cortex, and found that tuning similarity is the major determinant for voxelwise NCs. Furthermore, van Bergen and Jehee [22] systematically evaluated voxelwise NCs in human V1 to V3 and showed that the magnitude of NCs monotonically increases as tuning similarity increases. Furthermore, one recent study found that a multivariate classifier can exploit voxelwise NCs to decode population information [23]. Our recent work showed that the voxelwise noise correlations in general enhance the amount of information in a limited pool in human early visual cortex [24]. These results provide specific evidence supporting the existence of voxelwise TCNC, and suggest that a deeper understanding of how NC manifests in fMRI data is critical for studying probabilistic neural computation using multivariate fMRI data [22, 25].

In the present study, we combine MVPA and the voxel-encoding modeling approach to assess how the magnitude and form of NCs impact population codes in fMRI data. Similar to prior theoretical work in neurophysiology, we aim to derive the theoretical bound of the effects of voxelwise NCs on population codes in multivariate voxel responses. We assess the accuracy of population codes by MVPA and information-theoretic analyses. The voxel-encoding model used in this study allows us to systematically manipulate response parameters (i.e., voxel tuning) so as to examine NCs in different scenarios [26]. We first assess the quantitative relationship between decoding accuracy and the strength of NCs. We then directly calculate the amount of information as a function of NCs in a voxel population. Both methods demonstrate that the accuracy of population codes in fMRI data follows a U-shaped function as the strength of TCNC increases. Notably, all these analyses in voxel populations are compared against classical findings in neuronal populations. We show that the effects of NCs on population codes are strongly mediated by tuning heterogeneity in voxel populations.

## Materials and methods

**Fig 2:**
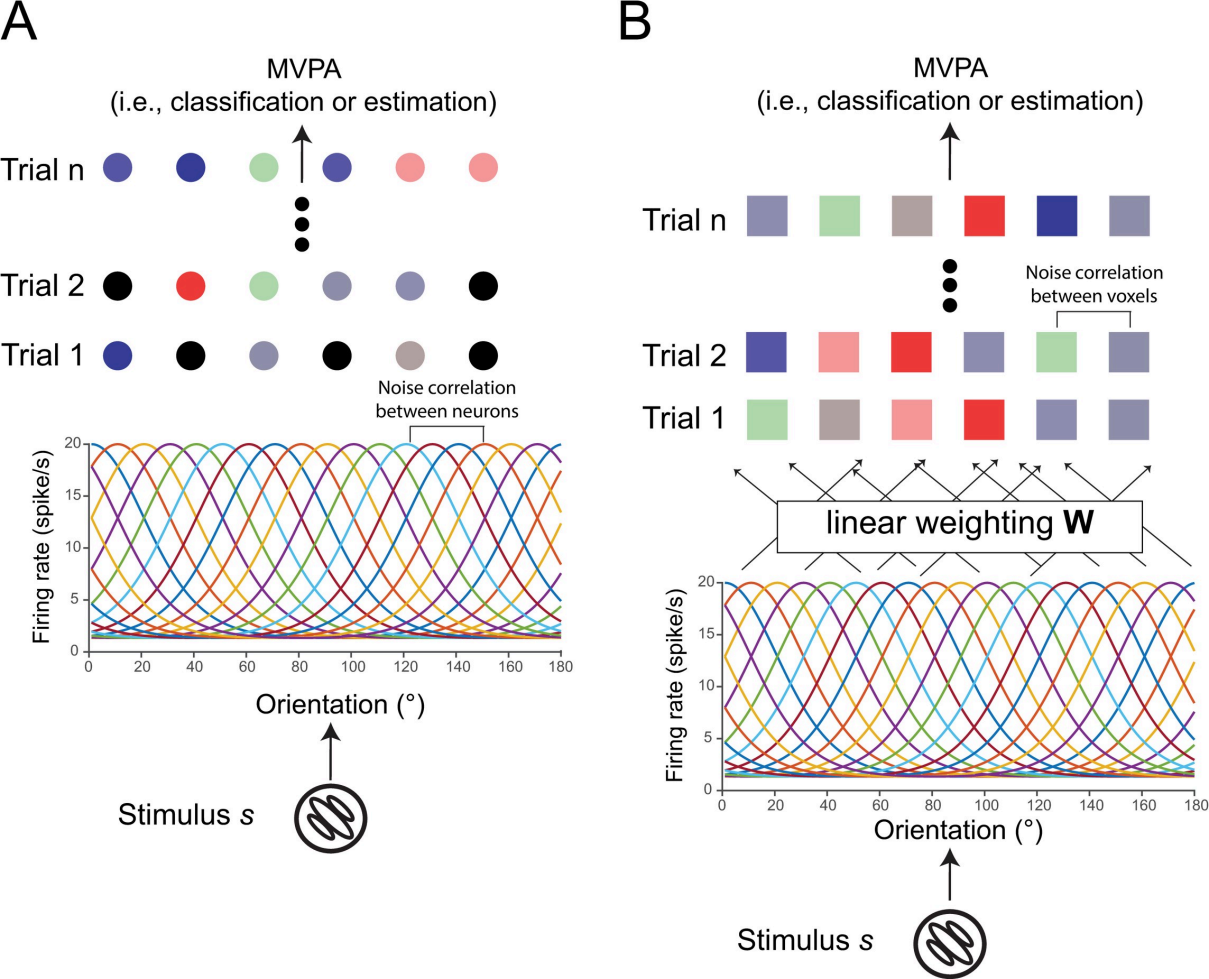
Neuron- and voxel-encoding models. The neuron-encoding model (panel A) proposes a neuronal population with orientation-selective tuning curves. Each neuron has Poisson-like response variance and the noise correlation between two neurons can be specified with different structures and strength (see Materials and Methods). The voxel-encoding model proposes a similar neuronal population and the response of a single voxel is the linear combination of the responses of multiple neurons. The noise correlation between two voxels can be specified using similar methods (see Materials and Methods). Note that voxelwise NCs can come from the response variability at both neuronal and voxel levels (see Fig 6). Using the neuron- and the voxel-encoding models, we can generate many trials of neuronal and voxel population responses and perform conventional MVPA on the simulated data. The goal is to examine multivariate decoding performance as a function of the NC structure and strength between either neurons or voxels.

Previous endeavors of brain decoding generally fall into two broad categories: classification of stimuli into discrete categories [27] and estimation of a continuous stimulus variable [28]. We thus evaluated the effect of NC in brain decoding in two tasks—a stimulus-classification task and a stimulus-estimation task. We will first introduce the simulation on a neuronal population and then specify the voxel-encoding model used to generate simulated responses of a voxel population (see Fig 2).

### Assessment of effects of noise correlations in neuronal populations

#### Neuron-encoding model

The neuron-encoding model assumes a pool of orientation-selective neurons whose preferred orientations are equally spaced between [1°, 180°]. We manipulated the number of neurons in our simulations. Similarly, all orientations throughout the entire paper are angles in degrees within [1°, 180°]. Tuning curves of the neurons can be described as:

$$g_{k}(s)=\alpha +\beta *e^{\gamma *(cos(\frac{\pi }{90}(s-\varphi_{k}))-1)}$$ (1)

where *g*_*k*_(*s*) is the tuning function of the *k*-th neuron. *s* is the stimulus. *φ*_*k*_ indicates the preferred orientation of the *k*-th neuron. *α* is the baseline firing rate, *β* controls the response range, and *γ* controls the width of the tuning curve. We set the parameter values *α* = 1, *β* = 19, and *γ* = 2, resulting in a tuning curve with the maximum firing rate at 20 spikes per second. This tuning curve is consistent with previous theoretical work [29] and empirical measurements in the primary visual cortex in primates [5].

Based on this setting, the mean of neuronal population responses given stimulus *s* can be represented by *G*(*s*) = [*g*_*k*_(*s*)]. However, empirically measured neuronal responses vary trial-by-trial. We posit that the mean of trial-by-trial population responses is *G*(*s*). We will detail the covariance in the following section.

#### Noise correlation and covariance

We proposed three types of NCs for neuronal data (see Table 1): angular-based tuning compatible noise correlation (aTCNC), curve-based tuning compatible noise correlation (cTCNC) and shuffled noise correlation (SFNC).

**Table 1:** List of symbols.

Symbol	Meaning
***NC***	Noise correlation
***SC***	Signal correlation
***fMRI*** ***MVPA***	Functional magnetic resonance imaging Multivariate pattern analysis
***TCNC***	Tuning-compatible noise correlation
***aTCNC***	Angular-based tuning-compatible noise correlation
***cTCNC***	Curve-based tuning-compatible noise correlation
***SFNC***	Shuffled noise correlation
***R*^*cTCNC*^**	Angular-based tuning-compatible noise correlation matrix
***R*^*aTCNC*^**	Curve-based tuning-compatible noise correlation matrix
***R*^*SFNC*^**	Shuffled noise correlation matrix
***c*_*neuron*_**	Noise correlation coefficient between neurons
***c*_*vxs*_**	Noise correlation coefficient between voxels
***c*_*homo*_**	voxel tuning heterogeneity coefficient
**W**	Linear weighting matrix from neuronal to voxel responses

Several theoretical studies assume the NC between a pair of neurons is an exponential function of the angular difference between their preferred orientations, here defined as angular-based tuning compatible noise correlation (aTCNC):

$$r_{ij}^{aTCNC}=e^{\left(-\frac{\left|\varphi_{i}-\varphi_{j}\right|}{L}*\frac{90}{\pi }\right)}$$ (2)

**Fig 3:**
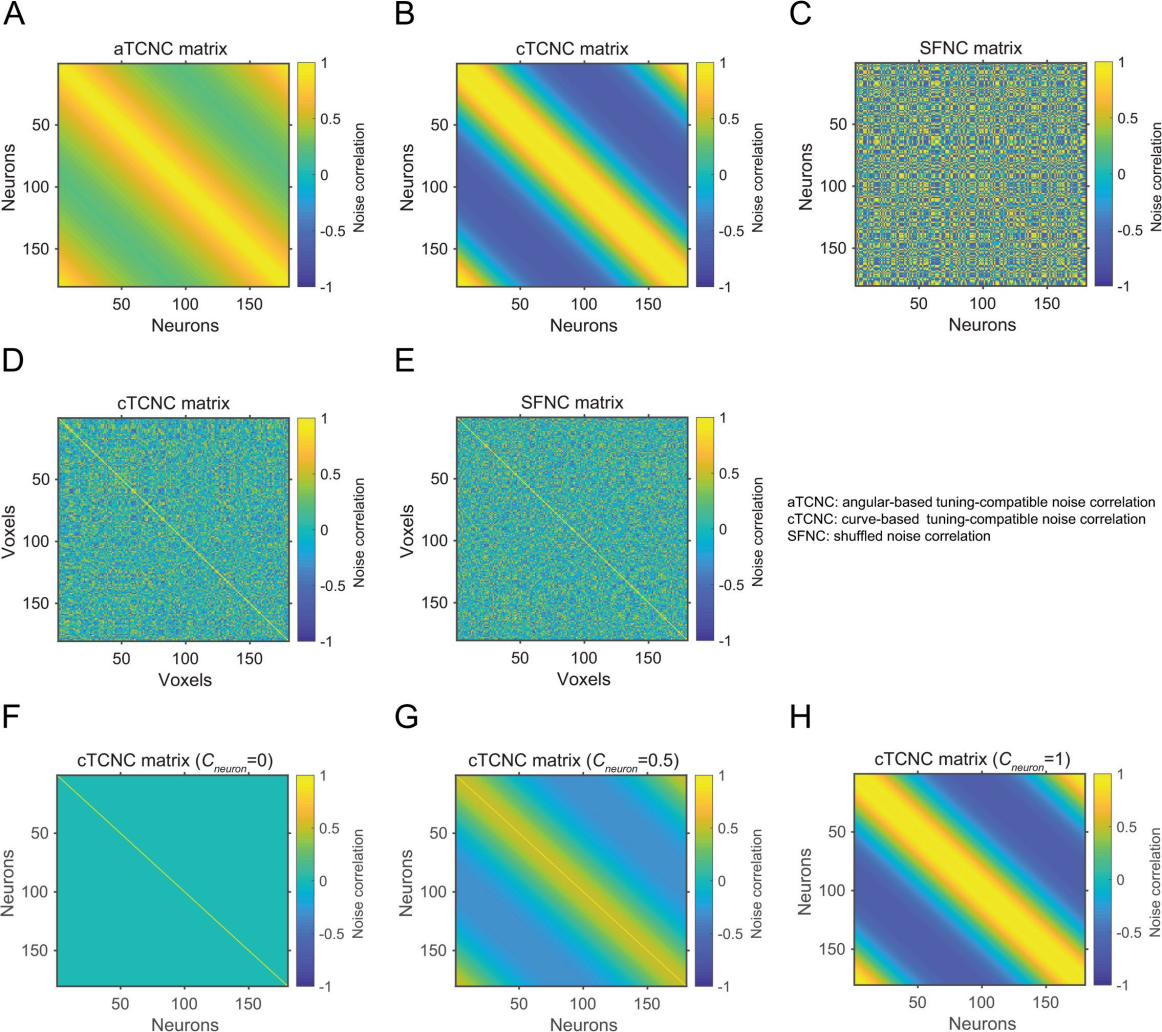
Example noise correlation matrices simulated in a neuronal (panels A-C) and a voxel population (D, E). In the neuronal population (180 neurons), the angular-based TCNC matrix, the curve-based TCNC matrix, and the SFNC matrix are illustrated from left to right. Neurons are sorted according to their preferred orientation from 1 to 180°. In the voxel population (180 voxels), the curve-based TCNC matrix and the SFNC matrix are illustrated. Note that we do not sort the voxels according to their tuning preferences. The NC coefficients (*c*_*neuron*_ or *c*_*vxs*_) are set to 1 in matrices from A-E. Panels F-H illustrate the cTCNC matrices with NC coefficient (*c*_*neuron*_) values 0, 0.5 and 1, respectively. Note that panels B and H are identical.

$r_{ij}^{aTCNC}$ is the NC between the *i*-th and the *j*-th neurons. φ_*i*_ and φ_*j*_ are their preferred orientations. This equation specifies that the NC between two neurons diminishes as their preferred orientations are farther apart. The parameter *L* controls the magnitude of such decay. We denote the correlation matrix as *R*^*cTCNC*^. Here we set *L* = 1 for simplicity. Ecker, Berens [29] has shown that the parametric form of NC and the value of *L* does not qualitatively change the result of the simulation, as long as the generated correlation matrix is positive definite. Note that by this definition aTCNCs are always positive (i.e., range 0~1, also see Fig 3A).

The second type is the curve-based tuning compatible noise correlation (cTCNC). In this case, the NC between a pair of neurons is proportional to their SC (i.e., correlation of their orientation tuning curves):

$$r_{ij}^{cTCNC}=(1-\delta_{ij})*corr\left(g_{i}(S),g_{j}(S)\right)+\delta_{ij},$$ (3)

where *δ*_*ij*_ is the Kronecker delta (*δ*_*ij*_ = 1 if *i* = *j* and *δ*_*ij*_ = 0 otherwise). **S** indicates all possible orientations between [1°, 180°], and $r_{ij}^{cTCNC}$ is the NC between the *i*-th and the *j*-th neurons. *g*_*i*_(**S**) and *g*_*j*_(**S**) are their tuning curves (see Eq 1). We denote *R*^*cTCNC*^ as the correlation matrix. Note that unlike aTCNCs, cTCNCs can be negative (see Fig 3B). Also, the key difference between cTCNC and aTCNC is that cTCNC does not rely on the functional form of tuning curves. In other words, cTCNC can be computed given irregular tuning curves, whereas aTCNC can be only computed from unimodal tuning curves. This is important for specifications of voxelwise NCs (see below).

In the third case, we shuffled the NCs between all pairs of neurons in *R*^*cTCNC*^ such that the rows and columns are rearranged in the same randomized order but the diagonal of the matrix is kept intact (Fig 3C). We term this type of NC as shuffled noise correlation (SFNC) since the correlation is no longer necessarily related to the neuronal tuning relations. We want to especially emphasize that here shuffling refers to untangling any relationships (e.g., linear relationship in aTCNC Eq 2 or cTCNC Eq 3) between noise correlations and tuning similarity (i.e., signal correlation), but noise correlations still exist. This is different from some studies in which multivariate responses data are shuffled across trials to completely eliminate noise correlations between voxels (i.e., all off-diagonal elements in a covariance matrix are 0) [30, 31]. Our case is similar to the situation that we randomly inject some noise correlations between voxels regardless of their tuning similarity. The correlation matrix of SFNCs is denoted as *R*^*SFNC*^. *R*^*SFNC*^ can serve as a comparison for *R*^*cTCNC*^ since shuffling does not alter the overall distribution of NCs in a neuronal population.

Furthermore, we assumed Poisson noise of spikes such that the response variance of a neuron is equal to the mean activity evoked by a stimulus.

$$\tau_{k}^{2}(s)=g_{k}(s)$$ (4)

where $\tau_{k}^{2}(s)$ is the response variance of the *k*-th neuron triggered by the stimulus *s*. Note that in this case the response variance is stimulus-dependent. The covariance between neurons *i* and *j* (*q*_*neuronij*_ as below) can be expressed as:

$${q_{neuron}}_{ij}=(1-\delta_{ij}){*c}_{neuron}*r_{ij}*\tau_{i}\tau_{j}+\delta_{ij}*\tau_{i}\tau_{j}$$ (5)

where *c*_*neuron*_ is a parameter that controls the strength of the neuronal NC. *τ*_*i*_ and *τ*_*j*_ are the standard deviation of responses of the two neurons (see Eq 4), respectively. *δ*_*ij*_ is the Kronecker delta. Given the covariance matrix **Q**_*neuron*_, we can express the population response noise distribution as:

$$e∼N(0,Q_{neuron}),$$ (6)

### Data simulation and multivariate pattern analysis

#### Stimulus-classification task

In the stimulus-classification task, we attempted to determine which of two stimuli were presented, based on the simulated neuronal population responses. We manipulated two independent variables: population size (i.e., the number of neurons) and NC strength (i.e., *c*_*neuron*_ in Eq 5). We built a linear discriminant using the Matlab function *classify*.*m*. The linear discriminant assumes that the conditional probability density functions *p* (**b** | *s* = *s*_1_) and *p* (**b** | *s* = *s*_2_) are both normally distributed with the same covariance and estimates the means and covariance from the training data. Here **b** is the vector of a population response in one trial (also see Eq 7). The classifier was trained on half of the data and tested on the other half.

For the neuronal populations, we attempted to classify two stimuli: *s*_*1*_ = 92°, *s*_*2*_ = 88°. The two stimuli were chosen to control the overall task difficulty (i.e., avoid ceiling and floor effects in classification accuracy). We set six pool size levels (i.e., 10, 20, 50, 100, 200, and 400 neurons) and six NC strength levels (i.e., *c*_*neuron*_ = 0, 0.1, 0.3, 0.5, 0.8, and 0.99). For each combination of a pool size and a *c*_*neuron*_ value and for each form of NC, we performed 100 independent simulations and then averaged classification accuracy values across simulations. To compensate for potential overfitting as the pool size increases, we set the number of trials for each stimulus to be 100 times the pool size. All data were equally divided into two independent parts for training and testing.

#### Stimulus-estimation task

In the stimulus-estimation task, neuronal responses in a trial were simulated for an orientation randomly chosen within [1°, 180°], and then a maximum likelihood estimator (MLE) was used to reconstruct the orientation value. Formally, given a population response pattern **b** in a trial, we attempted to find the stimulus *s* that maximizes the likelihood:

$$argmax_{x\in (1,180]}p(b|s)$$ (7)

**Fig 4:**
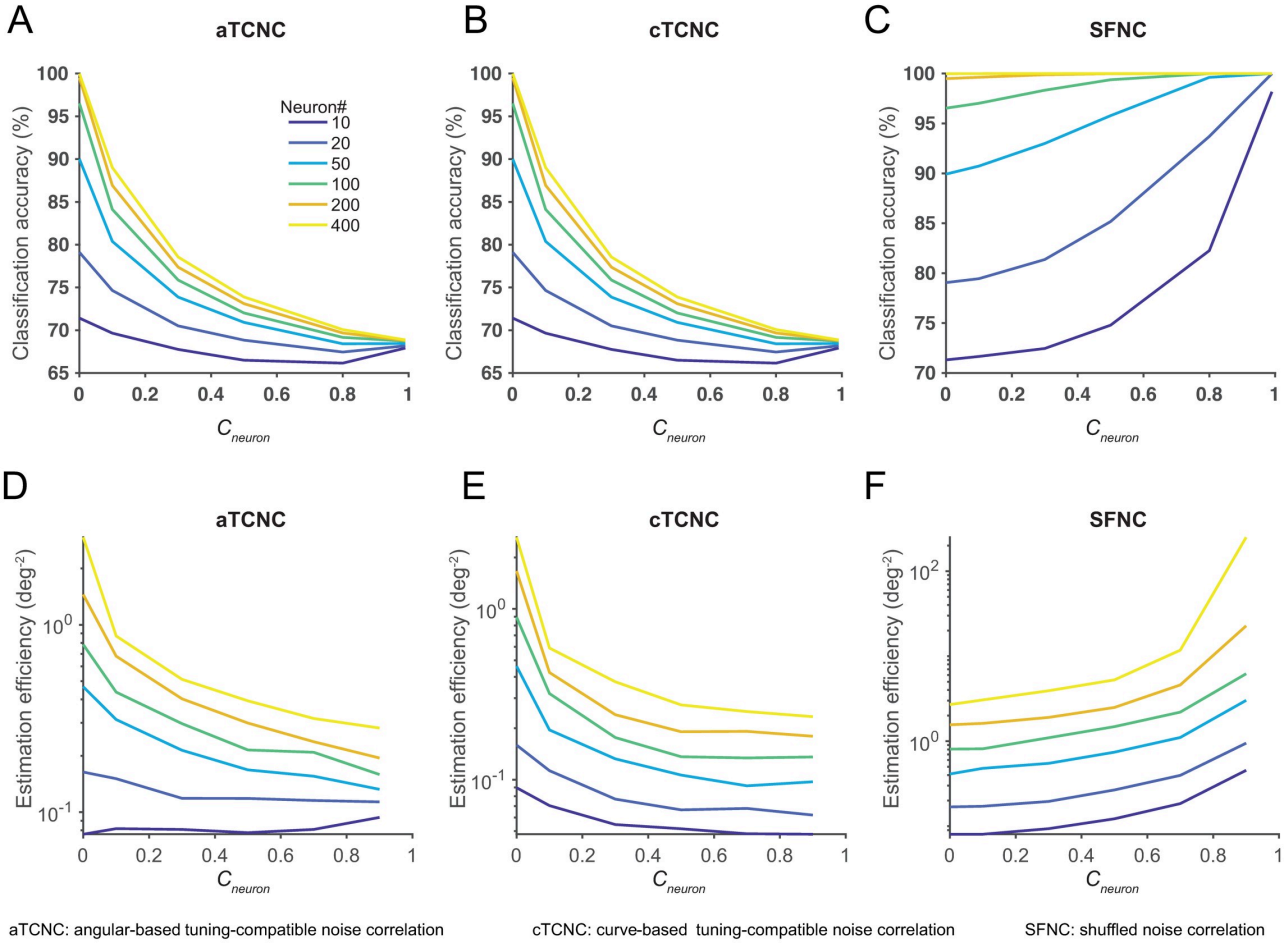
TCNCs impair population codes in a neuronal population. The multivariate classification accuracy (panels A-C) and maximum likelihood estimation efficiency (panels D-F) are depicted as a function of the magnitude of the aTCNC (panels A, D), TCNC (panels B, E) and the SFNC (panels C, F). Both classification accuracy and estimation efficiency decline as the strength of aTCNC and cTCNC increases. Conversely, increasing the strength of SFNC improves decoding accuracy.

**Fig 5:**
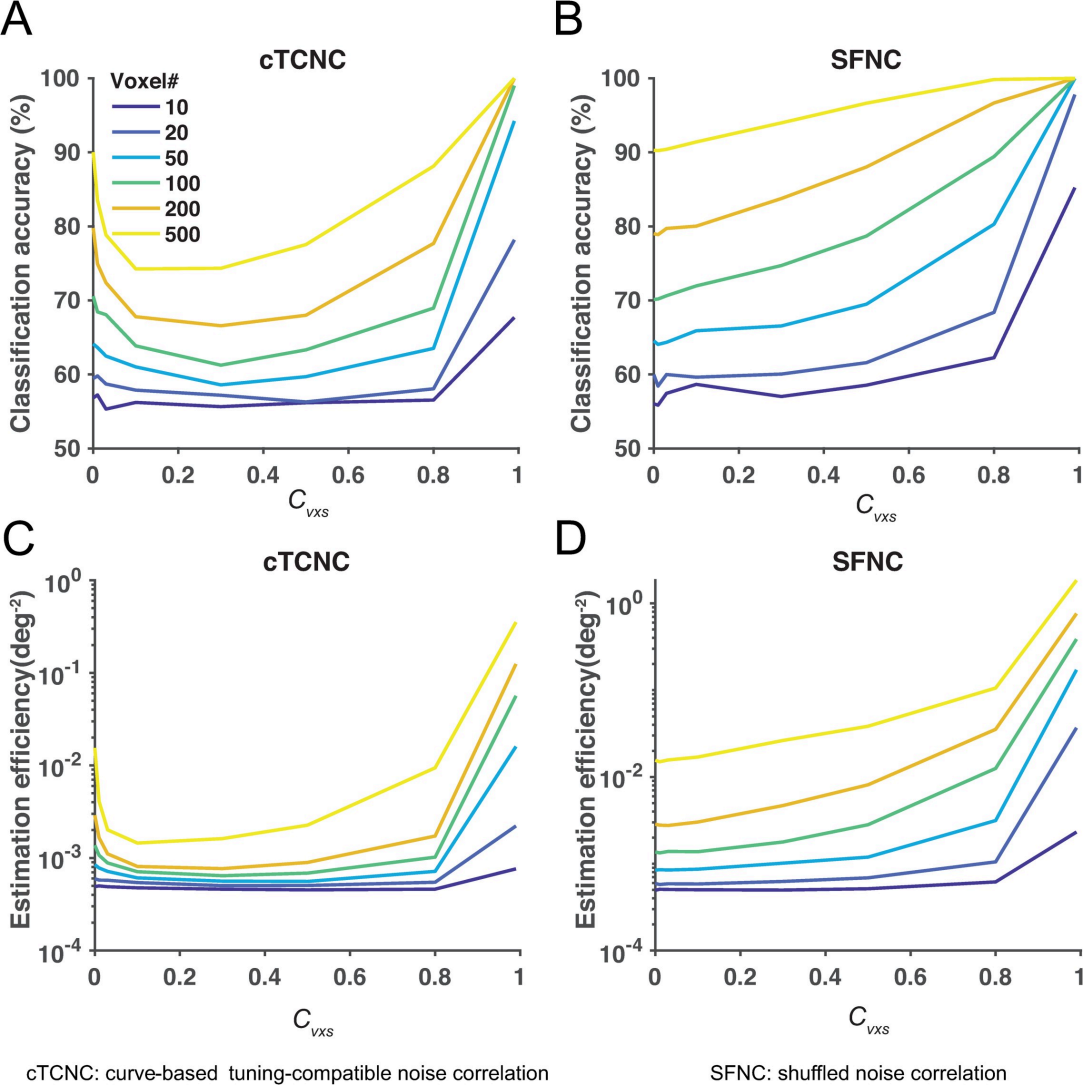
Decoding accuracy as U-shaped functions of cTCNCs in a voxel population. The multivariate classification accuracy (panels A, B) and estimation efficiency (panels C, D) are depicted as a function of the magnitude of cTCNCs (panels A, C) and SFNCs (panels B, D). Decoding accuracy exhibits U-shaped functions as cTCNCs increase. Similar to a neuronal population, SFNCs always improve decoding accuracy.

Note that the likelihood function has been introduced above as the neuron-encoding model (see noise distribution in Eqs 5 & 6). We numerically evaluated the likelihood of a pattern response **b** for each of 180 integer stimulus orientations (i.e., 1°–180°) and chose the orientation that yielded the maximum likelihood value. It is worth noting that, in contrast to classification, the MLE method does not involve any model training, and estimations were directly performed based on the known generative neuron-encoding model. We randomly sampled 1000 stimuli (i.e., 1000 trials) from [1°,180°] for decoding. The same pool size and *c*_*neuron*_ settings as in the stimulus-classification task were used. For each combination of a pool size and a *c*_*neuron*_ value, we calculated the mean circular squared errors (*MSE*_*circ*_) across all trials between the estimated stimuli (${\hat{s}}_{i}$) and the true stimuli (*s_i_*) across all trials:

$${MSE}_{circ}=\frac{1}{1000}\sum_{i=1}^{1000}{\left({\hat{s}}_{i}-s_{i}\right)}^{2},$$ (8)

where ${\hat{s}}_{i}$ is the estimated stimulus and *s*_*i*_ is the true stimulus in the *i*-th trial. We took the inverse of the *MSE*_*circ*_ as the estimation efficiency (see Figs 4 and 5). A higher estimation efficiency value indicates a more accurate estimation.

### Assessment of effects of noise correlations in voxel populations

#### Voxel-encoding model

The voxel-encoding model uses the same pool of orientation-selective neurons (i.e., 180 neurons with tuning curves defined in Eq 1) as in the neuron-encoding model. We further assume that the response of a voxel is the linear combination of all neurons in the neuronal population:

$$h_{i}(s)=\sum_{k=1}^{180}w_{ki}g_{k}(s),$$ (9)

where *h*_*i*_(*s*) is the tuning function of the *i*-th voxel. *w*_*ki*_ is the connection weight between the *k*-th neuron to the *i*-th voxel. We sampled *w*_*ki*_ from a uniform distribution:

$$w_{ki}∼uniform(0,0.01),$$ (10)

This range was used so that generated fMRI responses typically range between 0 and 10, and can be viewed as approximating units of percent blood-oxygen-level-dependent (BOLD) change. This is also consistent with the range of empirically measured fMRI responses in most studies.

The mean of voxel population response given stimulus *s* can be represented by **H**(*s*) = [*h*_*i*_(*s*)]. To express the trial-by-trial variation of voxel responses, we specify:

b=H(s)+e, (11)

Here, **b** represents the observed response across voxels on a trial (as might be obtained from a general linear model applied to fMRI data) and ***e*** represents the multivariate normal noise distribution:

$$e∼N(0,Q_{vxs}),$$ (12)

where **Q**_*vxs*_ is the covariance matrix between voxels, which will be detailed in the following section. It is noteworthy that we only calculate the voxel tuning curves as the weighted sum of the neuronal pool (Eq 9), but the voxel response variability does not only originate from neuronal response variability. If all voxel activities (including variability) are completely determined by a weighted sum of neuronal activities, the **H**(*s*) in Eq 11 should also be a random variable. However, in realistic fMRI data there are also other sources of voxel-level noise (e.g., thermal noise, head motion, see discussion) whose quantitative influences on voxel activity are difficult to delineate. Thus, we do not treat **H**(*s*) as a variable and instead assume an independent Gaussian noise (Eq 12).

#### Noise correlation and covariance

We evaluate two types of NCs for simulated fMRI data: cTCNC and SFNC. Note that we cannot evaluate aTCNC for voxel populations because voxel tuning curves here are irregular and not unimodal.

In the first case, we defined cTCNC using a similar method as Eq 3:

$$r_{ij}^{cTCNC}=(1-\delta_{ij})*corr\left(h_{i}(S),h_{j}(S)\right)+\delta_{ij},$$ (13)

where $r_{ij}^{cTCNC}$ is the NC between voxels *i* and *j*. Note that the cTCNC here is based on the tuning curves of two voxels (i.e., *h*_*i*_(**S**) and *h*_*j*_(**S**)), not two neurons. *δ*_*ij*_ is the Kronecker delta.

In the second case, SFNCs were generated using a similar method as in the neuron-encoding model—shuffling the rows and columns in *R*^*cTCNC*^, which is obtained in Eq 13.

We assume the response variances for different voxels (e.g., $\tau_{k}^{2}$ for the *k*-th voxel) follow a Gamma distribution:

$$\tau_{k}^{2}∼Gamma(u,v),$$ (14)

where *u =* 9, *v =* 0.33 are the scale and the shape parameters corresponding to a Gamma distribution with mean = 3 and variance = 1. Given the response variance of individual voxels and the NC between them, we can calculate the covariance between the *i*-th and the *j*-th voxels as:

$${q_{vxs}}_{ij}=(1-\delta_{ij}){*c}_{vxs}*r_{ij}*\tau_{i}\tau_{j}+\delta_{ij}\tau_{i}\tau_{i},$$ (15)

where *c*_*vxs*_ is the parameter that controls the strength of the voxelwise NCs. *τ*_*i*_ and *τ*_*j*_ are the standard deviation of responses of the two voxels (from Eq 14), respectively. *δ*_*ij*_ is the Kronecker delta. Given the covariance matrix **Q**_*vxs*_, we can finally generate voxel population responses using Eqs 11 & 12. Note that Eq 14 describes the variability of the response variance across voxels. The distribution of voxel population responses still follows a multivariate Gaussian distribution (Eq 12).

### Data simulation and multivariate pattern analysis

#### Stimulus-classification task

In the voxel-encoding model, we reduced the task difficulty and set the two stimuli as *s*_*1*_ = 80°, *s*_*2*_ = 100°. The motivation for changing task difficulty is to compensate for the higher noise level in voxel responses and avoid ceiling or floor effects in classification. We set six pool size levels (i.e., 10, 20, 50, 100, 200, and 500 voxels) and eight NC strength levels (i.e., *c*_*vxs*_ = 0, 0.01, 0.03, 0.1, 0.3, 0.5, 0.8, and 0.99). 10 independent simulations were performed. In each simulation, we assessed the classification accuracy for each combination of a pool size and a *c*_*vxs*_ value. Since the voxel tuning curves are determined by the linear weighting matrix **W**, in each simulation, we generated a new **W** for a given pool size. This ensures that we generated a new set of voxels in every simulation such that our conclusion is not biased by a particular choice of **W**. The **W** was kept constant across different *c*_*vxs*_ values such that classification accuracy values are directly comparable across different *c*_*vxs*_ values. For each stimulus, we simulated 1000, 1000, 1000, 1000, 2000, and 5000 trials for the corresponding pool sizes, respectively. We increased the number of trials for large pool sizes to avoid overfitting.

#### Stimulus-estimation task

In the stimulus-estimation task, we used the same pool size and NC strength settings as in the neuron-encoding model. We also performed 10 independent simulations and generated simulated responses to 1000 stimuli between [1°, 180°] in each simulation. Similar to above, for each simulation and each pool size, we recreated a linear weight **W** to create a new set of voxels, and kept the same **W** across *c*_*vxs*_ values. Similar to neuronal populations, the inverse of circular mean square error (Eq 8) was calculated to indicate the estimation efficiency. The estimation efficiency values were averaged across the 10 simulations.

### Simultaneously manipulating neuronal and voxelwise noise correlations

**Fig 6:**
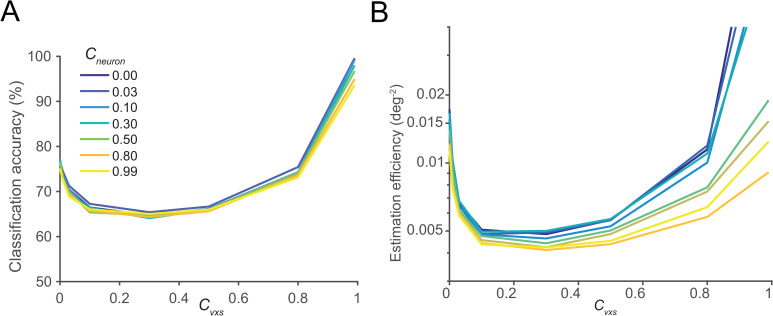
The impacts of neuronal and voxelwise cTCNCs on stimulus classification (A) and estimation (B). In both tasks, decoding performance exhibits U-shaped functions of the strength of voxelwise cTCNCs (i.e., *c*_*vxs*_). Neuronal cTCNCs (i.e., *c*_*neuron*_) have small impacts on classification accuracy, because the voxel-level noise primarily limits information. Neuronal cTCNCs have a more prominent detrimental effect in the estimation task. These results are consistent with the results when two levels of cTCNCs are manipulated independently.

In previous simulations, we either only manipulated the neuronal NCs in the neuron-encoding model or the voxelwise NCs in the voxel-encoding model. However, in realistic fMRI responses, voxel responses will inherit NCs from the neural level and will also include other sources of NC (such as head motion). To examine the interaction between neuron-level and voxel-level NCs on decoding accuracy, we simultaneously manipulated both neuronal and voxelwise NCs in the voxel-encoding model (Fig 6). In this simulation, we kept the same settings as the simulations above in the voxel-encoding model except for the following changes. First, we fixed the pool size to 200 voxels and manipulated the cTCNCs at the neuron level. We set eight cTCNC strength levels at the neuron level (same as in the neuron-encoding model). Second, in every trial of classification or estimation, we first generated a neuronal population response (i.e., responses for 180 neurons). Note that this generation takes into account neuron-level NCs. We then linearly transformed the neuronal population response into a voxel population response using the linear weighting matrix **W** (10 different **W** for 10 independent simulations), which yields the mean of the voxel population response. Finally, to generate the voxel population response observed on a given trial, we added voxel-level cTCNCs to the mean voxel population response, as we did in previous simulations.

### Differences between the neuron- and the voxel-encoding models

In this section, we summarize three key differences between the two encoding models proposed above. The biggest difference is that decoding is performed directly on simulated neuronal responses in the neuron-encoding model, but is performed on simulated voxel responses, which are linear combinations of the underlying neuronal responses, in the voxel-encoding model. Second, the NCs we manipulate are between neurons in the neuron-encoding model. In the voxel-encoding model, we either only manipulate the NCs at the voxel level (Fig 5) or the NCs in both neuron and voxel stages (Fig 6). In empirical fMRI studies, neuronal NCs are inaccessible; thus, the former case is more pertinent to realistic fMRI data analysis while the latter case provides theoretical insights. Third, we assume Poisson-like response variance for individual neurons in the neuron-encoding model, which is consistent with the previous theoretical work and empirical findings [1]. In this regime, the magnitude of response variance of individual neurons is stimulus-dependent. In the voxel-encoding model, we assume stimulus-independent additive Gaussian noise for voxels, consistent with one recent computational study [22].

### Information-theoretic analyses

**Fig 7:**
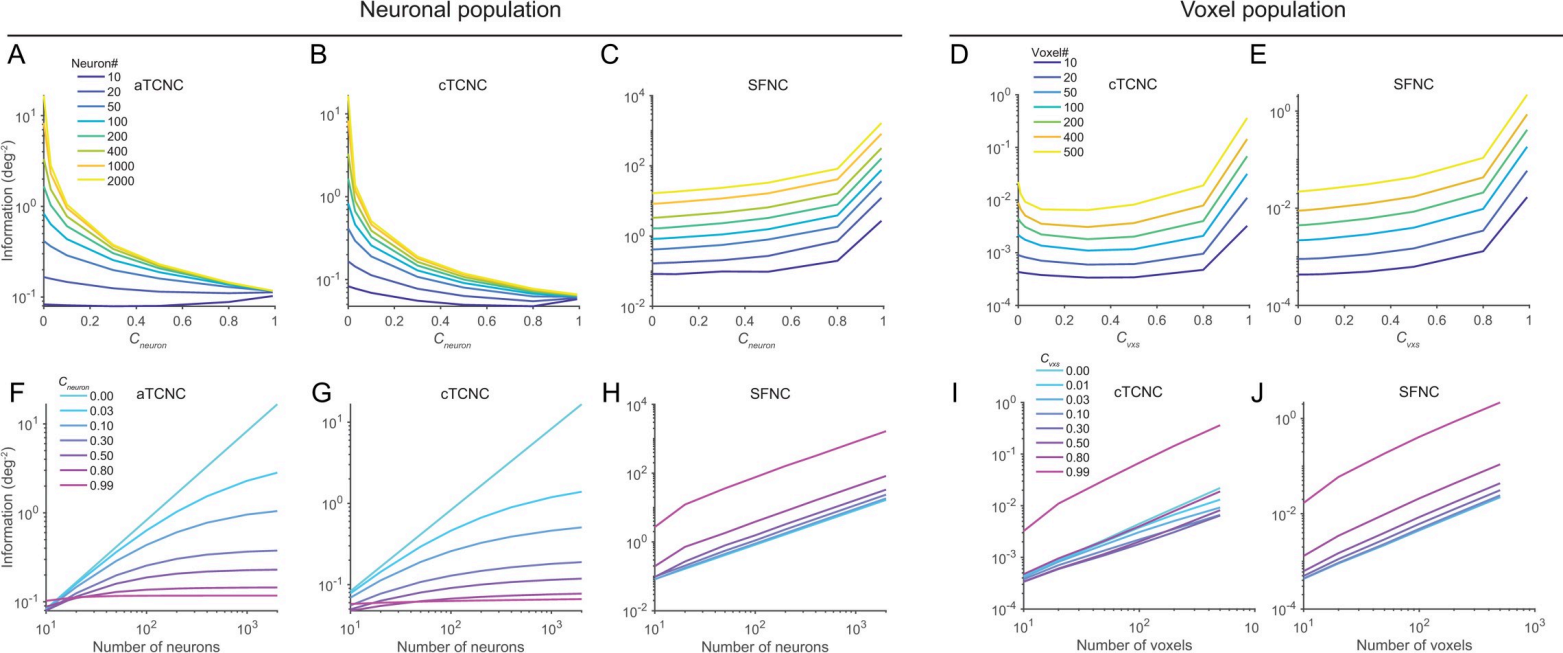
Amount of information in neuronal and voxel populations with diverse forms and strength of NCs. The upper and the bottom rows depict the amount of information as a function of increasing strength of NCs and the increasing number of units in the population, respectively. Panels A-C and F-H illustrate the amount of information in a neuronal population and correspond to Fig 4. Panels D-E and I-J illustrate the amount of information in a voxel population and correspond to Fig 5. Note that here we only illustrate the information in the stimulus-estimation task. We consider three types of NC—aTCNC (panels A, F), cTCNC (panels B, G), and SFNCs (panels C, H) in the neuronal population, as already shown in Fig 4. Similar treatments are performed for the voxel population, as shown in Fig 5. The calculation of information largely mirrors the decoding results shown in Fig 4 and Fig 5. Critically, the amount of information in the voxel population exhibits U-shaped functions of increasing strength of cTCNCs (panel D) and cTCNCs do not limit information as the number of voxels increases (panel I). These results clearly differ from the effects of aTCNCs (panels A, F) and cTCNCs (panels B, G) in the neuronal population.

We calculated Fisher information in the stimulus-estimation task (Fig 7) as it is one of the standard methods to quantify information in computational neuroscience [32]. Specifically, we used linear Fisher information and can be expressed as:

$$I(s)=f{\prime (s)}^{T}*Q^{-1}(s)*f\prime (s),$$ (16)

where **f**′(*s*) is the derivative of the mean population responses with respect to stimulus *s* and **Q (**i.e., **Q**_***neuron***_ or **Q**_***vxs***_**)** is the covariance matrix given stimulus *s*. Note that linear Fisher information can be calculated from both simulated neuron- and voxel-encoding models as long as the tuning curves and the covariance matrix are known. Notably, in neuronal data, complete Fisher information is stimulus-dependent because of the assumed Poisson noise distribution and the covariance matrix **Q** varies across stimuli. Note that linear Fisher information *per se* does not require the assumption of Gaussian variability and is suitable for any response distribution belongs to exponential family with linear sufficient statistics [30, 33]. But given the limited number of neurons or voxels recorded and the limited number of trials in empirical studies, the direct application of Eq 16 may contain bias and the analytical solution to correct the bias requires Gaussian assumption [31]. In the simulated voxel data, we assumed additive Gaussian noise and thus the covariance matrix **Q** is identical for all orientations (i.e., stimulus-invariant) and thus linear Fisher information is equivalent to complete Fisher information. In this paper, we simply denote both as “information”. We computed the averaged linear Fisher information for all 180 discrete orientations:

$$I=\frac{1}{180}\sum_{s=1}^{180}I(s),$$ (17)

where *s* is the stimulus. Note that theoretically linear Fisher information above only applies to the stimulus-estimation task or a fine-discrimination task. For a general classification task (i.e., classify two stimuli *s*_1_ and *s*_2_), especially for a coarse discrimination task, Eq 16 can be rewritten as the discrete format:

$$I={\left(\Delta f\right)}^{T}*Q^{-1}*\Delta f,$$ (18)

where Δ**f** = **f**(*s*_1_)−**f**(*s*_2_) is the population response difference for two stimuli and $Q=\frac{Q(s_{1})+Q(s_{2})}{2}$ is the covariance matrix. This metric by definition is not Fisher information and typically called “linear discriminability” [34]. To avoid confusion in terminologies, we denoted both metrices as “information” in this paper as they indicate the quality of population codes in the two tasks respectively. In the main text, we only show the information in the stimulus-estimation task (Fig 6).

Fisher information can be converted into a stimulus discrimination threshold Δ*θ*.

$$\Delta \theta =2*\frac{\Phi^{-1}(PC)}{\sqrt{I}}$$ (19)

where *I* is information, *PC* is percent of correct with respect to the threshold, and **Φ**^−1^ is the inverse cumulative normal function.

### Varying voxel tuning heterogeneity

**Fig 8:**
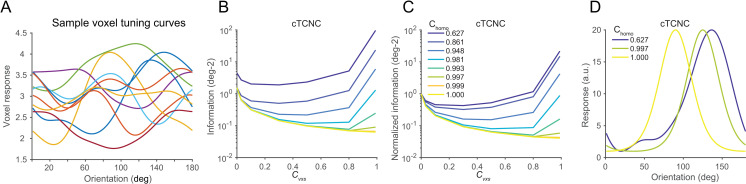
Interaction between cTCNC and tuning heterogeneity on population codes. 500 voxels were simulated (see Materials and Methods). A larger value of c_*homo*_ indicates more homogeneous voxel tuning curves. Note that the simulated voxel tuning curves are identical to neuronal tuning curves when c_*homo*_ = 1. Panel A illustrates some sample tuning curves of the simulated voxels. Due to the uncertain neuron-to-voxel connections (i.e., linear weighting matrix **W**), the endowed voxel tuning curves also exhibit irregular forms. Panels B and C illustrate the raw and normalized amount of information as a function of cTCNC under different tuning heterogeneity levels. The raw information is normalized to the condition when *c*_*vxs*_ = 0 (panel C). As voxel tuning homogeneity increases, the shape of the functions changes from U-shaped to monotonically decreasing. Panel D illustrates sample voxel tuning curves with different heterogeneity levels.

To illustrate the effect of tuning heterogeneity, we performed an additional analysis on the voxel-encoding model (Fig 8). In this analysis, we calculated the amount of information in the stimulus-estimation task after making the following modifications. First, we fixed the voxel pool size to 500. Second, we introduced the heterogeneity coefficient (c_*homo*_) that controls the voxel tuning heterogeneity. The key to manipulating heterogeneity is to adjust the linear weighting **W** from neuronal to voxel responses. For each voxel, we first randomly selected one neuron from all 180 neurons and assigned c_*homo*_ as the linear weight for this neuron. The weights for other neurons were then assigned by random numbers between 0~1 scaled by (1- c_*homo*_) (i.e., (1- c_*homo*_)*rand in Matlab). For example, if c_*homo*_ = 1, the voxel tuning curve is homogeneous and identical to the neuronal tuning curve chosen in the first step; if c_*homo*_ = 0, the voxel tuning curves are heterogeneous as it is the linear combination of all other neurons with random weights (see Fig 8A). Third, one might speculate that differences in results across neuronal and voxel simulations might due to the absolute response range. In the neuron-encoding models, the response range of neuronal tuning curves is between [1, 20] spikes per second whereas the voxel tuning curves are smaller than 10. To control this absolute difference in the response ranges, we normalized the range of voxel tuning curves to [1, 20] (see Eq 1, also see scaled voxel tuning curves in Fig 8D compared to Fig 8A). Note that in this case voxel response amplitude is larger than that in the previous voxel simulation (<10). Larger response amplitudes will result in overall higher information, we thus also scaled the voxel variance 40 times (i.e., the mean of Gamma distribution in Eq 14) to keep the comparable signal-to-noise levels in voxel responses.

### Simulating noise correlations based on real fMRI data

We also simulated noise correlations based on measurements from real fMRI data. van Bergen and Jehee [22] found that, in an orientation fMRI experiment, noise correlations between voxels is an exponential function of their signal correlations:

$$r_{ij}^{cTCNC}=a*e^{\beta (corr(h_{i}(S),h_{j}(S))-1)}+\lambda ,$$ (20)

where *α* = 0.14, *β* = 1.99, *λ* = 0.09. These values are drawn from the curve fit in ref. [22]. Note that this equation specifies that the magnitude of voxelwise NC is between [0.09, 0.23]. We repeated the voxel encoding model simulation by replacing Eq 13 with Eq 20. To test the effects of the magnitude of NC, we also set *α* = 0.9, such that the maximal NC can reach 0.99 and rerun the simulation.

### Code availability

All code is available at https://github.com/ruyuanzhang/noisecorrelation

## Results

### 1. Effects of noise correlation in neuronal and voxel populations on both stimulus estimation and classification

In the first part, we will show the effects of noise correlation on population codes in both neuronal and voxel populations. We simulated multivariate responses with three and two forms of noise correlation in neuronal and voxel populations respectively. We performed two brain decoding tasks—a stimulus-classification task and a stimulus-estimation task. In the stimulus-classification task, a linear classifier was trained to categorize evoked population responses into one of two discrete orientation stimuli. In the stimulus-estimation task, a maximum likelihood estimator (MLE) was used to reconstruct the continuous orientation value based on the population response in a trial. Both tasks are two routinely used forms of MVPA in the literature [17, 18, 27].

#### 1.1 Tuning-compatible noise correlations between neurons impair decoding accuracy in multivariate neuronal responses

Before examining the effect of TCNC in a voxel population, we first attempted to replicate the classical findings in a standard neuronal population. In the simulation of neuronal population responses, all neurons shared the same tuning curve except that their preferred orientations were equally spaced in the continuous orientation space (Fig 2A, also see Materials and Methods). We manipulated three types of NCs between neurons—angular-based tuning-compatible noise correlation (aTCNC), curve-based tuning-compatible noise correlation (cTCNC), and shuffled noise correlation (SFNC). The first one is also called ‘limited-range correlation’ in neurophysiological literature [3, 12]. aTCNCs are based on the angular difference between the preferred orientations of two neurons (Fig 3A). Specifically, we defined that the strength of NC between two neurons follows an exponential decay function (Eq 2) of the absolute angular difference between their preferred orientations. This approach has often been used to establish population coding models [12, 29, 35]. The second type, cTCNC, is based on the similarity between the tuning curves (i.e., SC) of two neurons (Fig 3B). We defined that the sign and magnitude of cTCNCs are the same as and proportional to the SC between two neurons. This is consistent with empirical measurement in electrophysiology [12, 29, 35]. Note that both aTCNC and cTCNC are related to the tuning similarity between two neurons since the larger angular difference between the two neurons’ preferred orientations, the less their tuning curves are correlated. SFNCs served as a control condition and were generated by randomly shuffling the cTCNCs between neurons (Fig 3C, see Materials and Methods) such that they had no relationship with the tuning properties of neurons.

We manipulated two variables of the neuronal population—the pool size (i.e., the number of neurons) and the strength of NCs between neurons (i.e., *c*_*neuron*_, see Fig 3F, 3G and 3H). For every combination of pool sizes and NC strength levels, we simulated population responses in many trials and performed the MVPA decoding (i.e., classification and estimation) on the simulated population responses.

Results indeed replicated the findings from previous theoretical work [12]. aTCNCs and cTCNCs impaired decoding performance in both tasks: the classification accuracy (Fig 4A and 4B) and the efficiency of the MLE (Fig 4D and 4E) declined as the strength of aTCNCs and cTCNCs increased. The only exception is that the overall decoding accuracy is a U-shaped curve for small pool size (N = 10). This is consistent with the information analyses below. Decoding performance always rose as the strength of SFNCs increased. This result is similar to the finding in [36]. We will explain this phenomenon in the later sections.

Note that in real experiments, one cannot easily manipulate the generative structure of voxel activity. The manipulation of SFNC here should be seen as a data analysis method that removes the relationship of NC to tuning similarity while keeping the marginal distributions of NCs identical (i.e., the items in the SFNC matrix are identical to those in the TCNC matrix but rearranged across rows and columns). Also, here we explore properties of a population containing either pure TCNCs or pure SFNCs, two extreme cases in theoretical modeling. The correlation structure in empirical data is likely in between these two extremes.

#### 1.2 Decoding accuracy as U-shaped functions of tuning-compatible noise correlations in multivariate voxel responses

We next turned to examine the impact of NC on population codes in fMRI data. We simulated responses of a voxel population using a voxel-encoding model (Fig 2B) and attempted to perform the classification and estimation tasks.

We again manipulated two types of NCs—cTCNC (Fig 3D) and SFNC (Fig 3E). The cTCNCs here are similar to above except that they are between voxels rather than neurons. Similarly, cTCNCs here are defined with respect to the similarity of their orientation tuning curves. SFNCs were also generated by randomly shuffling the cTCNCs between voxels (see Materials and Methods). Note that we cannot parametrically derive aTCNCs for voxels as we did for neurons since unlike unimodal orientation tuning curves of cortical neurons, orientation tuning curves of voxels might be irregular (i.e., multimodal) due to the mixing of multiple neural populations in a voxel’s activity (see Eq 9 and Fig 8A). We will return to this point in a later section.

We found that the decoding performance exhibited U-shaped functions of the increasing amount of cTCNCs: both classification accuracy (Fig 5A) and estimation efficiency (Fig 5C) first declined and then rose in both tasks. This is puzzling since the predominant view in neurophysiology regards cTCNCs as detrimental but here we demonstrated that cTCNCs improve population codes. SFNCs in general improved the decoding accuracy, similar to the effect observed in a neuronal population.

Note that the manipulation of the strength or structure of noise correlations in simulated data is only an approach in theoretical modeling, suggesting no feasible means that can be used to manipulate realistic data.

#### 1.3 Simultaneously varying neuronal and voxelwise noise correlations

In empirical fMRI studies, we can only measure voxelwise NCs but the sources of these NCs are unclear. One important source might be neuronal NCs because neuronal NCs could propagate to the voxel level if voxel responses are believed to be the aggregation of neuronal responses. However, fMRI data might also involve other MRI-specific noises (hemodynamic fluctuations, thermal noise, head motion, etc.). It is thus reasonable to assume that voxel-level NCs reflect the combinations of neuronal and other voxel-level NCs. Systematically disentangling these factors would be a useful direction for future experimental studies, but here we can at least derive some theoretical expectations using our analytical framework. In previous analyses, we only manipulated either the NCs between neurons or the NCs between voxels. We next manipulate both neuronal and voxelwise cTCNCs in the voxel-encoding model.

We repeated the classification and the estimation tasks on a voxel population (see Materials and Methods for details). Results showed that increasing neuronal-level cTCNCs had a small impact on classification accuracy and the change in the classification accuracy values was primarily determined by voxel-level cTCNCs. This is because we attempted to decode two stimuli (*s*_*1*_ = 80°, *s*_*2*_ = 100°) based on simulated fMRI responses. But this is a very easy task if we classify the two stimuli directly from neuronal responses (i.e., reach 100% correct ceiling, also see Materials and Methods). Thus, classification accuracy here is primarily bottlenecked by the noise at the voxel level not the neural level. Note that these results are contingent on the noise structure and strength assumed at both processing stages.

In the stimulus-estimation task, neuronal cTCNCs dampened estimation efficiency and voxelwise cTCNCs impact estimation efficiency as U-shaped functions. Both results are consistent with the previous results when two levels of NCs were manipulated independently. Our results provide a theoretical demonstration to our knowledge that how both neuron-level and voxel-level noises manifest in fMRI data.

### 2. Results of information-theoretic analyses explain the effects of noise correlation on population codes

In the second part, we will show how to use information-theoretic analyses to support the simulation results above. Especially, we want to highlight the unit tuning heterogeneity as a mediator for the effect of NCs in a population. Unit tuning heterogeneity also acts as the key factor to explain the differential effects of cTCNC in neuronal and voxel populations.

#### 2.1 Amount of information echoes decoding accuracy in population codes

Above analyses focused on assessing the population codes from the decoding perspective (i.e., MVPA), the approach that almost all previous fMRI decoding studies used. Here, we propose an alternative approach—directly calculate the amount of Fisher information for the stimulus-estimation task or linear discriminability for the stimulus-classification task. They have been used as the standard metric for information coding in computational neuroscience [32, 37]. For the estimation task, Fisher information indicates the minimal amount of variance that any unbiased decoder can possibly achieve. For the classification task, linear discriminability measures the magnitude of separation of two multivariate response distributions. It is also called a variant of linear Fisher information for a classification task [1]. For simplicity, we termed both metrics as “information” as they both indicate the accuracy of population codes with respect to the two tasks.

The analysis of information has three major advantages over the conventional MVPA approach. First, in theory two approaches might lead to consistent results as more information in a population usually leads to a higher decoding accuracy. But their relationship is nonlinear. Classification accuracy can reach the floor (e.g., 50% for binary classification) and ceiling (i.e., 100%) but the amount of information has a relatively broad range thus more sensitive to population codes. For example, as we will show, information in a standard neuronal population saturates as a function of pool size given the presence of aTCNCs and cTCNCs but not SFNCs (Fig 7F, 7G and 7H). Information in a voxel population keeps increasing as the pool size increases (Fig 7F–7J). These conclusions cannot be easily derived from decoding analyses per se. Second, here we used the optimal Bayesian decoder for the stimulus-estimation task and the linear discriminant for the stimulus-classification task. In most classification studies, researchers used some machine learning methods, such as support vector machine, logistic regression, linear regression. It still remains unclear whether these decoders are statistically optimal. The decoding results above might due to the particular decoders we use. In contrast, the assessment of information is not related to the assumptions or efficacy of any particular decoder. Third, most decoding methods so far employed discriminative modeling approach. Calculation of information here takes into account data generative processes. As such, calculation of information should be a more principled way to assess the accuracy of population codes.

We calculated the amount of information in both the neuronal and the voxel populations (see Materials and Methods) as functions of pool size and NC strength. Results largely mirrored the previous decoding results. In the neuronal population, we replicated the key signatures of detrimental effects of TCNCs: the amount of information saturated as the pool size increased given the presence of aTCNCs (Fig 7F) and cTCNCs (Fig 7G) but not SFNCs (Fig 7H). Also, the amount of information declined as the magnitude of aTCNCs (Fig 7A) and cTCNCs increased (Fig 7B). The only exception is the curves as a slight U-shaped function for small pool size (*N* = 10, Fig 7A and 7B). This pool size is very rare in realistic data. Note that the different curves in Fig 7A and 7B will converge when NC coefficient is 1, indicating that the information saturate very quickly as pool size increases (Fig 7F and 7G). The overall declining pattern was reversed as the magnitude of SFNCs increased (Fig 7C). In the voxel population, the amount of information always increased as the pool size expanded in both cTCNCs (Fig 7I) and SFNCs (Fig 7J) conditions. Similar to the decoding results, the amount of information exhibited U-shaped functions as the magnitude of cTCNCs (Fig 7D) increased and always grew as the magnitude of SFNCs (Fig 7E) increased.

The overall amount of information in voxels are much lower than that in neurons, because of the difference in signal-to-noise ratio of the two measurements. This is consistent with empirical finding that the sensitivity of a single neuron can predict or even surpass behavior but decoding accuracy in fMRI is always lower than behavioral performance. Fisher information can also be converted into an orientation discrimination threshold. For example, the linear Fisher information of 100 neurons is 1.5 deg^-2^ when cTCNC is 0 (Fig 7B). This is equivalent to 1.1 deg threshold corresponding to 75% accuracy (Eq 19). This threshold is close to the behavioral threshold reported in ref. [38]. Similarly, the threshold is 20.1 deg for 100 voxels when cTCNC is 0. This is also consistent with the coarse orientation discrimination task in fMRI [27].

#### 2.2 Voxel tuning heterogeneity and pool sizes explain the effect of tuning-compatible noise correlations on population codes

Why do TCNCs manifest differently in neuronal and voxel populations? We reason that the neuron-to-voxel transformation (i.e., linear weighting **W**) might be the key factor that alters the effect of TCNCs. Unlike the homogeneous neuronal tuning curves (i.e., same width, amplitude, and baseline, and only preferred orientations vary), voxel tuning curves might be heterogeneous or have diverse tuning widths and amplitudes (Fig 8A). This is due to the uncertain distribution (i.e., the weighting matrix **W** in Eq 9) of orientation-selective neurons within a voxel. Even though individual neurons follow a uniform bell-shape tuning property, the aggregation of them can produce tuning functions with diverse forms. Because of the tuning heterogeneity, TCNCs do not limit information anymore. The effect of tuning heterogeneity has been studied in some previous theoretical work [16, 29, 36] (see more details in discussion).

To further substantiate the interaction effect between tuning heterogeneity and NCs on population codes, we manipulated the degree of voxel tuning heterogeneity and the strength of cTCNCs in the voxel population. The amount of information was calculated as a function of these two variables (Fig 8B and 8C). Results showed that the amount of information follows U-shaped functions if voxel tuning is highly heterogeneous (i.e., c_*homo*_ = 0.03 in Fig 8). However, as the voxel tuning becomes progressively homogeneous (i.e., c_*homo*_ increases to 1), cTCNCs become more and more detrimental for information coding, which is consistent with the results obtained in a standard neuronal population (Fig 7B). These results suggest that the cases of cTCNCs and SFNCs in neurons represent two extreme cases where NCs impair or enhance information. But there exists a continuum of possible scenarios that could lead to mixture of detrimental and beneficial effects. The observed U-shaped function is one example. It is likely that the tuning-compatible NCs indeed impose detrimental effects to some extent, but the effects are mitigated by tuning heterogeneity. If tuning functions become more heterogenous, the two antagonistic effects together produce a U-shaped function.

**Fig 9:**
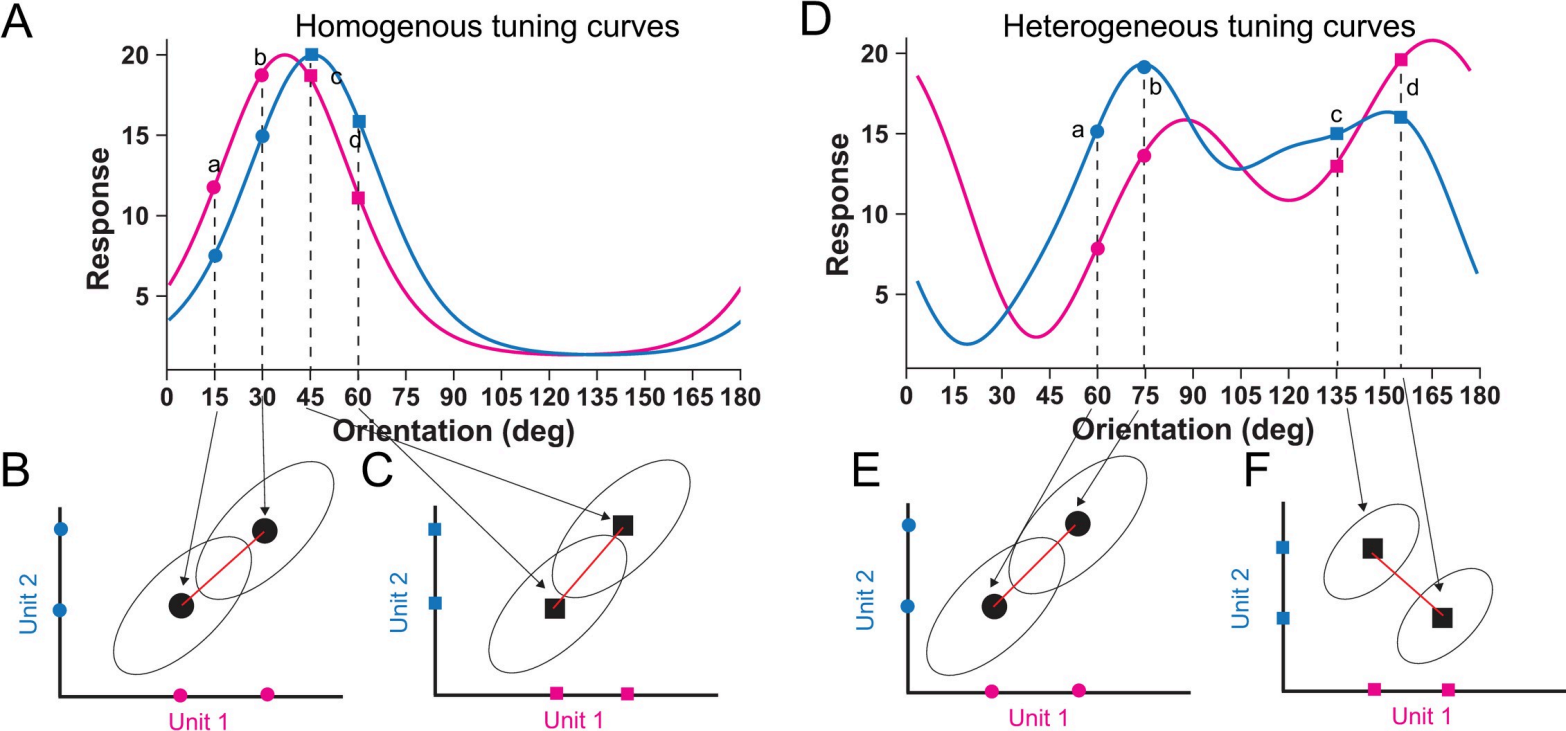
The detrimental effects of TCNCs and the beneficial effects of tuning heterogeneity. Panel A illustrates the scenario of homogeneous tuning curves of two units. Panels B and C depict the cases of classifying stimuli a and b and stimuli c and d, respectively. In both panels B and C, the noise correlation is detrimental. The dots or squares on the x- and y-axes indicate the mean responses of the two units towards the two stimuli. Panels D-F are similar to Panels A-C but illustrate the scenario of heterogeneous tuning curves. The noise correlation between the two units is detrimental to the classification of stimuli a and b, but beneficial to the classification of stimuli c and d. Panels D-F show how tuning heterogeneity can mitigate the detrimental effect of TCNC.

We provide an intuitive example that illustrates the detrimental effects of TCNC and the beneficial effects of tuning heterogeneity in Fig 9. If the tuning curves of two units are homogenous and similar (Fig 9A), by definition they should also have a high positive NC (i.e., TCNC). Because of high tuning similarity, the signal vectors (the red vectors in Fig 9B and 9C) connecting the mean of responses towards the two stimuli always align with the direction of noise correlation (i.e., the directions of the distribution ellipses). This type of noise correlation has been termed “differential correlation” and been shown to be detrimental because a decoder cannot well differentiate signal and noise. If the tuning curves are heterogeneous (Fig 9D), there can still exist a positive noise correlation between the two units (i.e., TCNC). The positive NC impairs the classification of stimuli a and b in Fig 9E. However, the positive NC is actually beneficial when classifying stimuli c and d, which is markedly different from the case in Fig 9C. We can imagine that the more heterogeneous tuning curves are, the higher the likelihood that the scenario of Fig 9F will occur.

Our results also highlight the complexity of quantifying information in a population and suggests that the influences of NCs must be systematically probed using a wide and systematic range of parameters. Taken together, we demonstrated that unit tuning heterogeneity is at least one of the key factors that mediates the contribution of cTCNCs in both neuronal and voxel populations.

#### 2.3 Simulating noise correlations based on realistic fMRI data

**Fig 10:**
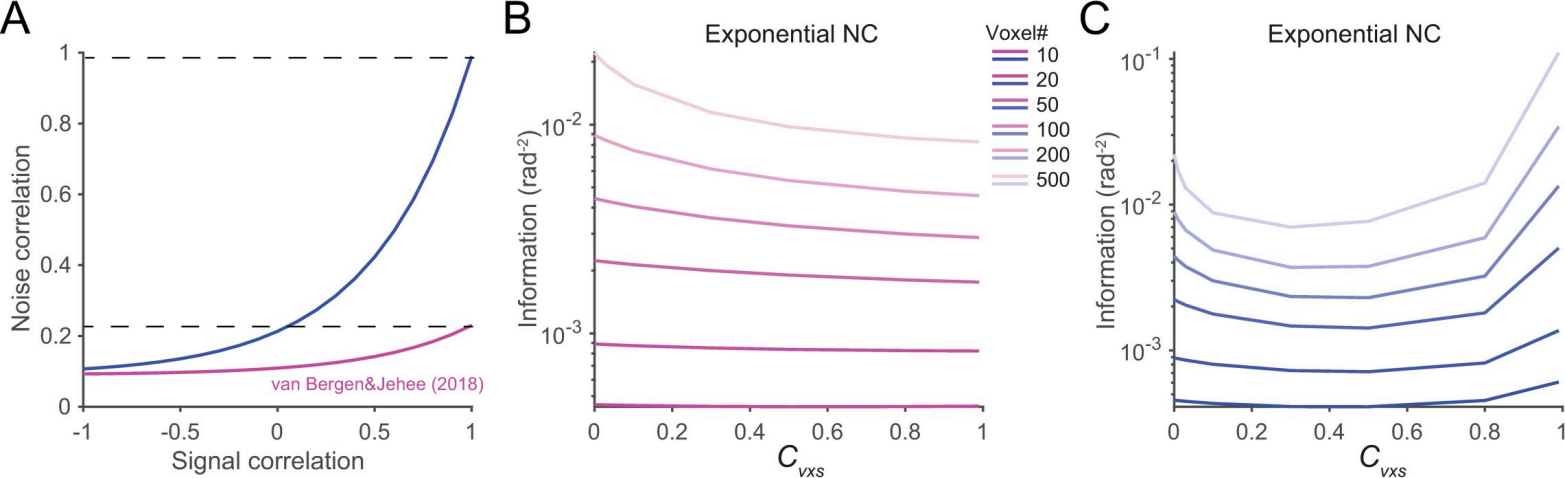
Simulation of voxel population responses based2 on realistic fMRI data. ***A***. noise correlation as an exponential function of signal correlation. The magenta curve is estimated from van Bergen and Jehee [22] and we scale it to increase the magnitude of noise correlations (i.e., the blue curve). ***B*,*C***. simulated effects of noise correlations in the regimes of the magenta and the blue curves, respectively. Noise correlations reduce the amount of information because the overall magnitude is relatively week (i.e., the magenta curve). We again observe U-shaped functions if the overall magnitude of noise correlations are large (i.e., the blue curve).

We next attempted to simulate voxel responses based on the relationship between SC and NC measured from realistic fMRI data. van Bergen and Jehee [22] found that the noise correlation between two voxels follow an exponential function of their signal correlation (the magenta line in Fig 10A). We used the exponential function estimated from that dataset to simulate our results (see Materials and Methods for details). We found that increasing voxel noise correlation coefficient reduces the amount of information. We speculate this is because the maximal noise correlations can only reach ~0.23 even if *c*_*vxs*_ reaches 1 such that only the declining part of the U-shaped functions is observable. To test this, we amplified the exponential function (the blue line in Fig 10A) and set maximal noise correlations to ~0.99 (see Materials and Methods). We again found the U-shaped functions consistent with the results above. This result also suggests that systematic theoretical analysis can reveal the full spectrum of possible consequences induced by noise correlations in population codes, which might be difficult to obtain by looking at individual datasets.

As shown in these simulations, the effects of NCs might be highly dependent on the choices or exact values of parameters (tuning width, NC strength etc.) in the data. Whether NCs in realistic data improve or impair information is still an open question and to-date there lacks direct evidence in this field. Our recent work found that the voxelwise NCs indeed enhance information in human V1 [24]. Future studies need to further test the effects of NCs in other stimulus features and cognitive tasks.

#### 2.4 The dominance of a fraction of good noise correlations explains the effect of shuffled noise correlations on population codes

Besides the beneficial effect of TCNCs, we turn to another interesting finding—SFNCs improve population codes in both neuronal and voxel populations. At first glance, this seems surprising since it suggests that decoding accuracy can be improved by, if possible, randomly creating some NCs between voxels. Here we want to highlight an intuitive explanation—some beneficial NCs might override the effects of detrimental NCs and disproportionally enhance decoding accuracy in the conventional multivariate analysis.

**Fig 11:**
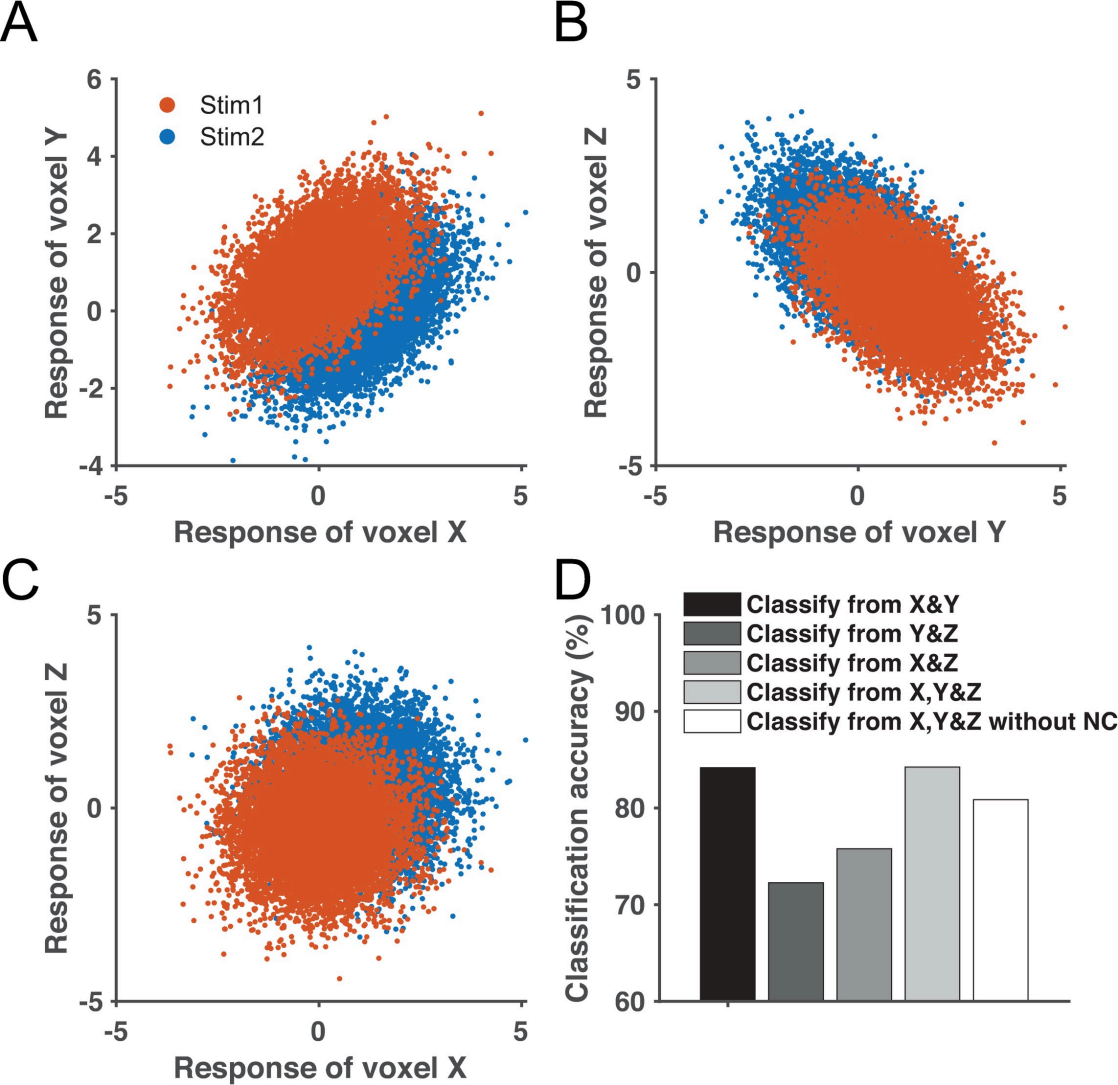
A three-voxel simulation illustrating the disproportional benefits of good covariance in multivariate decoding. Panel A illustrates the trial-by-trial responses of voxels X and Y towards two stimuli. The covariance structure of X and Y enhances classification accuracy. Similarly, panel B illustrates that the covariance structure of voxels Y and Z impairs classification. Voxels X and Z have no systematic NC (panel C). Panel D depicts the classification accuracy based on population responses of X and Y, Y and Z, X and Z, and all three units. We also include a situation where we set all NCs among three units to 0 and keep other settings the same (i.e., X, Y&Z without NC). We add this condition because in most empirical scenarios we are interested in comparing a population code with and without NCs. The beneficial and detrimental effects of the covariance structures in panels A and B do not cancel each other if all three voxels are combined.

We simulated a simple three-voxel scenario for a classification task to illustrate this effect (Fig 11). The NC between voxels X and Y improves classification (Fig 11A), while the NC between voxels Y and Z impairs classification (Fig 11B). The correlation between X and Y, and the correlation between Y and Z are identical in magnitude but with opposite signs. However, when all three voxels are aggregated, the contributions of the two opposite NCs do not cancel out each other and the overall decoding performance is still improved by the positive NC between X and Y, regardless of the negative NC between Y and Z. Importantly, classification accuracy on X, Y, and Z with NCs is higher than the scenario in which there are no noise correlations. These results demonstrate that, as long as there exists some voxels whose NCs are beneficial, these good NCs may dominate in the contribution to information. In other words, the most informative units may disproportionally enhance population codes and override the negative effects of other “bad” NCs (Fig 11). Note that this example only illustrates a possible scenario that the effects of good NCs can override bad NCs, but does not suggest this finding always holds. Precise estimations of effects of NCs require formal calculations of information.

This principle provides an intuitive explanation for the effects of SFNCs on decoding. In the scenario of SFNCs, the NC between a pair of voxels bears no resemblance to their tuning similarity. Given many voxels in a population, the NCs could be beneficial for some voxels (e.g., Fig 11A) or detrimental (e.g., Fig 11B) for others. The total information is the aggregation of both beneficial and detrimental effects. As we show in Fig 11, given the presence of both beneficial and detrimental NCs, the former type may persist, since a decoder can adjust its weights to fully utilize the beneficial NCs and minimize the effects of detrimental NCs. As the pool size increases, it becomes increasingly likely that a small fraction of units are assigned NCs that benefit decoding, resulting in overall enhanced decoding performance for the entire population.

## Discussion

Characterizing the effect of noise correlation on population codes has attracted much attention in the past years as it is related to several key topics (e.g., probabilistic computation, uncertainty) in neuroscience research [1, 25]. But the majority of relevant studies are confined to the field of neurophysiology. On the other hand, fMRI can measure many responsive units in the brain but most prior fMRI studies only employed MVPA to evaluate population codes. MVPA accuracy, however, is merely a coarse description of population codes and the precise quantitative relationship between the voxelwise NCs and population codes still remains unclear. Here, we conducted a series of theoretical analyses to systematically examine how NCs with different forms and strength influence MVPA accuracy and the amount of information in multivariate fMRI responses. We made two major observations: (1) decoding accuracy and the amount of information follow U-shaped functions of cTCNCs in a voxel population and this effect is mediated by voxel tuning heterogeneity and pool sizes; (2) assuming that the sign and magnitude of NCs between voxels are irrelevant to voxel tuning similarity, increasing the NCs will in general improve population codes. These results suggest that tuning heterogeneity of voxels helps distribute information across voxels and such that noise can be averaged out by increasing voxel number in a pool (i.e., information will not be limited as pool size increases). Furthermore, the comparisons against a standard neuronal population demonstrate that the effect of NC in both neuronal and voxel populations can be understood within a unified computational framework related to tuning heterogeneity.

### Noise correlation in neural processing

The effects of NC on the capacity of the neural population codes have been investigated in various studies over the past two decades [14–16, 29], leading to somewhat mixed results. Early results in neurophysiology suggest that cTCNCs could be detrimental [12], but later studies suggest that the results may be more complicated, depending on the detailed configurations of neural codes. There are regimes where the cTCNCs could be beneficial [16, 29, 36]. Wilke and Eurich [36] found that making the magnitude of the positive correlations irrelevant to tuning similarity benefits neural codes. They further provided an intuitive argument on why noise correlations that have no direct relationship to unit tuning might increase coding capacity. Their results are consistent with our findings on the benefit of SFNCs. We would like to emphasize that the results of theoretical work on this issue highly depend on the detailed specifications of the correlation structure in a population. For example, we can add a small term proportional to the outer product of the tuning curve derivative (i.e., so-called ‘differential correlations’) in Eq 5. Differential correlations have been shown to limit information in a population [30]. But the existence and amount of differential correlations in human fMRI data still need to be further explored. We can only conclude that our findings hold true in our simulated correlation structures.

The assumption of homogeneous tuning curves in early theoretical work is apparently not realistic because in the primate brain it has been known that the shape of tuning curves varies drastically across neurons. Such tuning heterogeneity removes TCNCs’ limitation on information. This theoretical implication has been also corroborated by an empirical study on orientation decoding in primate V1 [39]. Most importantly, the principle of tuning heterogeneity applies both neuronal and voxel populations.

Ecker, Berens [29] derived a mathematical foundation for the effects of tuning heterogeneity, pool size, and TCNC on population codes, built up the earlier work by Sompolinsky, Yoon [15]. In some recent work, the effects of NCs can be understood by investigating the projections of signals on each eigenvectors of the covariance matrix. The signature of so-called “differential correlations” appears as the large projections on the first few eigendimensions [38, 40]. Here, we extend previous work and demonstrate several novel aspects of NC in both neuronal and voxel populations. First, previous theoretical work in neurophysiology primarily focused on estimation tasks (but see [30]) while the majority of neuroimaging research focused on classification tasks. We compared both tasks in both populations. Second, previous work only analyzed one type of TCNC (i.e., aTCNC in theoretical work) and we systematically compared three types of NC in both populations. Third, we manipulated cTCNC at both neuronal and voxel activity stages to approximate more realistic interactions between neuronal and fMRI responses. These efforts not only enrich existing work in neurophysiology, but also provide a theoretical foundation to understand the effects of NCs in multivariate fMRI data.

### Quantifying information in fMRI data

In this paper, we focused on two routinely used perceptual tasks—the stimulus-estimation task and the stimulus-classification task. Stimulus estimation is equivalent to a very fine-discrimination task as it needs to discriminate the true stimulus value from nearby stimuli in the feature pace. It is primarily determined by Fisher information. Binary classification is more similar to coarse discrimination as it depends on the distance of the representations of two stimuli. In classification tasks, linear discriminability is a better measure than Fisher information [34, 41–43].

Given our finding that voxelwise noise correlations have substantial influence on population codes, it may be informative to measure the magnitude and form of noise correlations in empirical fMRI measurements. This is for three reasons. First, noise correlations must be taken into account in order to build an optimal decoder. From the Bayesian perspective, a statistically optimal decoder must have the full knowledge of how data are generated so that the generation process can be inverted (also see next section). Second, if the goal in a given fMRI study is to maximize decoding accuracy, it is an open question whether noise correlations should be kept or removed (e.g., whitening) in fMRI preprocessing. Because Fisher information is a U-shaped function of the strength of noise correlations, cognitive processes (e.g., attention, learning) that reduce noise correlations can either improve or impair decoding, depending on the exact structure of noise correlations in empirical data. Thus, reduced voxelwise NCs does not necessarily imply better population codes in fMRI. One still needs to either directly assess decoding accuracy or stimulus information (e.g., Fisher information).

Third, if the goal is to understand the effect of some modulatory factors (e.g., attention) on population codes, noise correlations might reflect important aspects of how this modulation is achieved by the brain [44].

### Towards a generative understanding of multivariate fMRI responses

In contrast to the enthusiasm for characterizing generative processes of stimulus-evoked responses in neurophysiology, only a few studies have performed generative modeling on fMRI data [22, 45]. Conventional neuroimaging approaches use MVPA to decode information from fMRI data [17, 18]. However, in recent years, people have increasingly realized the limitations of MVPA as a discriminative modeling approach, in which one seeks to estimate the probability *p*(stimulus | response). Rich representational information might be buried by merely examining decoding accuracy [19].

From a probabilistic modeling perspective, understanding the generative computation in the brain is equivalent to deriving the joint probability *p*(response, stimulus), which is equivalent to calculating to *p*(response | stimulus) × *p*(stimulus) according to Bayes’ theorem. Current voxel-encoding modeling approaches seek to characterize the mean of the likelihood term, *p*(response | stimulus), in the sense of characterizing the computations by which a stimulus produces population responses in the brain. However, the full likelihood function *p*(response | stimulus) also requires characterizing the covariance between voxels. Calculating the full likelihood or joint distribution of responses and stimuli can provide important insight into the probabilistic computation in the human brain [45].

### The nature of noise correlations in fMRI data

Although fMRI can naturally measure the activity of many units in the brain, the investigation of NCs in fMRI data has just begun recently. Exploring this issue in fMRI data is, however, non-trivial and we summarize the related issues as follows.

First, the definition of “noise correlation” in fMRI research is still under debate. The well-accepted definition of “noise correlation” in electro-physiology is the correlation of trial-by-trial responses between two neurons given the repeated presentation of the same stimulus. This definition emphasizes stimulus-evoked responses. In this paper, we strictly follow this definition and assume voxel responses as trial-by-trial responses estimated from the standard general linear model. This is also called “beta series correlation” in some fMRI literature [46]. In contrast, one recent study defined the noise correlation between two voxels as their resting-state functional connectivity or background functional connectivity during a task [23]. In theory, these definitions deviate from the conventional definition in computational neuroscience and their quantitative relationship remains unclear. Only one recent study suggested that resting-state functional connectivity is highly correlated with the trial-by-trial response correlation at the whole-brain level [47]. Future studies need to examine the relations between resting-, task-based functional connectivity, and trial-by-trial variation of responses at the individual voxel level.

Second, the sources of noise correlations in fMRI data are still unclear. On one hand, the conventional term “noise correlation” itself is somewhat misleading since, as shown in this paper, response variability can contain a substantial amount of stimulus information. In other words, response variability is not purely “noise” and might reflect some critical aspects of how neurons process stimulus or task structure [48].

In this paper, we assume the existence of tuning-compatible noise correlations between voxels. While the existence of such correlations has found some support [22], it is less clear what the causes of such correlations are. There are at least three types of factors that could contribute to such trial-by-trial variation of voxel activity. First, the variability of underlying neuronal activity can propagate to the voxel level, causing variations in voxel BOLD signals. For example, it has been shown that fMRI orientation decoding can be explained by coarse-scale orientation preference maps [49] and neuronal noise correlations are presumably present in this scenario. A second type of factor consists of global brain/cognitive signals, such as arousal, wakefulness, etc. These factors have been shown to modulate noise correlations in primates [3]. A third type of factor is non-neural noise arising in MRI data acquisition processes, such as cardiac- and respiratory-related noise, head motion, image reconstruction artifacts, etc. These types of noise should be carefully considered and ideally removed in data pre-processing, as they may otherwise lead to incorrect neuroscientific interpretations. Perhaps the most pernicious type of noise is head motion, which, due to its nature, may induce spatially structured noise and confer noise correlations between voxels that may share similar tuning profiles. How to best model and control for the influences of head motion is still an active topic in fMRI research. In addition, it remains a challenge for future studies to develop procedures for identifying and differentiating the effects of different types of noise.

We would like to offer two practical suggestions for empirical fMRI studies. First, although TCNC can improve stimulus information and noise in acquisition procedures might produce TCNC, this does not mean that noise is good: fMRI researchers should still make concerted efforts to minimize the magnitude of non-neural noise during data acquisition and pre-processing. Second, although researchers cannot directly manipulate neuronal noise correlations in conventional human experiments, researchers can readily manipulate cognitive states (e.g., attention) and quantify their effects on noise correlations. This approach has not yet been extensively applied in human studies, but may reveal unique neural mechanisms of cognitive processing that cannot be addressed by the conventional MVPA approach.
